# Mobile Technology Utilization Among Patients From Diverse Cultural and Linguistic Backgrounds Attending Cardiac Rehabilitation in Australia: Descriptive, Case-Matched Comparative Study

**DOI:** 10.2196/cardio.9424

**Published:** 2018-06-26

**Authors:** Ling Zhang, Ding Ding, Lis Neubeck, Patrick Gallagher, Glenn Paull, Yan Gao, Robyn Gallagher

**Affiliations:** ^1^ Sydney Nursing School University of Sydney Sydney Australia; ^2^ Charles Perkins Centre University of Sydney Sydney Australia; ^3^ Sydney School of Public Health University of Sydney Sydney Australia; ^4^ School of Health and Social Care Edinburgh Napier University Edinburgh United Kingdom; ^5^ Cardiology Department St George Hospital Kogarah Australia

**Keywords:** cultural and linguistic diverse, cardiac rehabilitation, technology, mobile technology, information technology

## Abstract

**Background:**

Barriers to attending cardiac rehabilitation (CR), including cultural and linguistic differences, may be addressed by recent technological developments. However, the feasibility of using these approaches in culturally and linguistically diverse patients is yet to be determined.

**Objective:**

This study aims to assess the use of mobile technologies and features, as well as confidence in utilization across patients speaking different languages at home (ie, English, Mandarin Chinese, and a language other than English and Mandarin [other]) and are both eligible and physically suitable for CR. In addition, the study aims to determine the sociodemographic correlates of the mobile technology/feature use, including language spoken at home in the three groups mentioned above.

**Methods:**

This is a descriptive, case matched, comparative study. Age and gender-matched patients speaking English, Mandarin and other languages (n=30/group) eligible for CR were surveyed for their mobile technology and mobile feature use.

**Results:**

‘Participants had a mean age of 66.7 years (SD 13, n=90, range 46-95), with 53.3% (48/90) male. The majority (82/90, 91.1%) used at least one technology device, with 87.8% (79/90) using mobile devices, the most common being smartphones (57/90, 63.3%), tablets (28/90, 31.1%), and text/voice-only phones (24/90, 26.7%). More English-speaking participants used computers than Mandarin or “other” language speaking participants (*P*=.003 and .02) and were more confident in doing so compared to Mandarin-speaking participants (*P*=.003). More Mandarin-speaking participants used smartphones compared with “other” language speaking participants (*P*=.03). Most commonly used mobile features were voice calls (77/82, 93.9%), text message (54/82, 65.9%), the internet (39/82, 47.6%), email (36/82, 43.9%), and videoconferencing (Skype or FaceTime [WeChat or QQ] 35/82, 42.7%). Less Mandarin-speaking participants used emails (*P*=.001) and social media (*P*=.007) than English-speaking participants. Speaking Mandarin was independently associated with using smartphone, emails, and accessing the web-based medication information (OR 7.238, 95% CI 1.262-41.522; *P*=.03, OR 0.089, 95% CI 0.016-0.490; *P*=.006 and OR 0.191, 95% CI 0.037-0.984; *P*=.05).

**Conclusions:**

This study reveals a high usage of mobile technology among CR patients and provides further insights into differences in the technology use across CALD patients in Australia. The findings of this study may inform the design and implementation of future technology-based CR.

## Introduction

Cardiac disease is a leading cause of morbidity and mortality worldwide [1]. Despite advancements in treatment and secondary prevention, the recurrence rate of cardiac events remains high [2], especially among specific sociodemographic groups, such as patients from culturally and linguistically diverse (CALD) backgrounds [3]. Cardiac rehabilitation (CR), a structured program of exercise and risk reduction education and counseling designed to promote healthy living with heart disease, effectively supports secondary prevention [4]. In addition, CR has been shown to reduce overall and cardiovascular-related morbidity and mortality, as well as hospital readmissions and length of hospital stay [5-11].

Despite established health benefits, CR remains underutilized. Globally, attendance rates remain as low as 15%-30% after a cardiac event [12-14], which, in fact, are attributed to provider-level barriers, such as the limited availability of services and inadequate referral, as well as patient-level characteristics, including old age, being female, and low socioeconomic status [14-20]. For example, people of CALD background are underrepresented in CR services [6,15,19,21,22]. Besides general barriers to CR, CALD patients experience unique challenges, such as limited English language proficiency, which render them less likely to be referred [21,23]. Transport difficulty [23], financial issues, and misperception of CR [23,24] are additional barriers to using the services once referred.

Of note, low attendance in CR among CALD patients is a characteristic of Western countries. Australia comprises an increasingly heterogeneous population. In 2015, approximately 6.7 million (28.2%) of the total Australian population were born outside of Australia [25]. At present, 1 in 5 Australians speaks a language other than English at home, of which Mandarin, Italian, and Arabic are the most common [26]. Chinese is one of the most rapidly growing CALD groups and has doubled in the past decade, currently constituting 2.2% of the Australian population [25]. A recent meta-analysis reported that Chinese living in Western countries have poorer short-term survival outcomes after a cardiac event [27] compared with Caucasians, which could be attributed to poor disease self-management [28]. In addition, Chinese immigrants are documented to be underserved by the current healthcare system because of incongruence between needs for support and available healthcare services such as CR services [29-31].

The ubiquity of mobile phones and advancements in mobile technology have facilitated the advent of new preventive delivery strategies which supplement center-based CR services to expand capacity. Contemporary mobile technology-based CR aims to monitor physical function, promote medication adherence, manage lifestyle, and provide health education to aid individuals manage their cardiac conditions [32,33]. The emerging evidence reveals that these programs could potentially attain similar benefits compared with center-based CR in decreasing risk factors and mortality in patients with coronary heart disease (CHD) [34]. In addition, the mobile technology-based CR could reach traditionally “hard-to-reach” populations, as delivery is not constrained by language, time, or transportation [33,35-37]. Furthermore, mobile technology-based CR is cost-effective for both service providers and patients [38], as it can save up to 80% of travel costs for patients compared with center-based CR [37].

Despite this promising potential, little investigation has been conducted on the utilization of mobile technology and the feasibility of the mobile technology-based CR in patients. In fact, no study has assessed how CALD patients might differ in their use of mobile technology and related features compared with other patients. Perhaps, comprehending the utilization of technological devices and mobile features, as well as the factors related to the use of these technologies among CALD patients, would facilitate the identification of CALD patients who might benefit from the mobile technology-based intervention.

This study aims to assess the relative use of mobile technologies and features, as well as confidence in utilization across patients speaking different languages at home [ie, English, Mandarin Chinese, and a language other than English and Mandarin (other)] and both eligible and physically suitable for CR. In addition, the study aims to determine the sociodemographic correlates of the mobile technology/feature use, including language spoken at home in the three groups mentioned above.

## Methods

### Study Design

This descriptive, case-matched, comparative study collaborated with a larger study that investigated cardiac patients’ use of mobile technology and variations among age groups after adjusting for education, employment, and confidence in using the mobile technology. The larger study surveyed 282 English-speaking CR patients on the mobile technology use in nine hospital and community sites across metropolitan and rural New South Wales, Australia [39]. This study enrolled 30 English-speaking patients from the large study to match with a separated Mandarin-speaking group and reported on multilingual groups recruited from this study site that has not been published previously.

### Setting

The study was conducted in a metropolitan teaching hospital in South Eastern Sydney Local Health District (New South Wales, Australia). The selected health district represented approximately 12% of the New South Wales population; with 37% born in countries outside of Australia and 27% in a non-English-speaking country, the selected health district comprised the most diverse population [40]. Of all, China-born residents constituted the largest proportion of the population from a non-English-speaking background, followed by people born in Greece and Indonesia. Of those born in countries outside of Australia, approximately 10% reported that they either do not speak English well or at all [41].

### Sample Eligibility and Exclusion Criteria

We recruited a stratified and matched convenience sample in this study. The inclusion criteria were as follows: (1) the presence of a cardiac diagnosis, such as angina, myocardial infarction (MI), ischemic heart disease (IHD), valve surgery, coronary artery bypass graft (CABG), or percutaneous coronary intervention (PCI), the absence of severe comorbidities, and physically suitable to be referred to the exercise-based group CR program; and 2) could speak and understand adequate English or Mandarin for consent and questionnaire processes. Patients with a neurocognitive disorder were excluded from the study. We matched each Mandarin-speaking patient by age (within ±5 years) and gender with a patient from the other two linguistic backgrounds to minimize the demographic variability across groups. Furthermore, the sample size was predetermined to be 30 per group per previous study protocols [42,43].

### Measurement

We used a previously developed checklist to collect sociodemographic and clinical data [44]; the information comprised participants’ age, gender, country of birth, ethnicity, home language, education, marital and employment status, and admission diagnosis.

Then, we developed the survey based on previously validated and used questionnaires where possible; the list of the most common devices (ie, smartphones, computers, and tablets) and mobile features (ie, browsing the internet, text messages, emails, and social media) was based on previously determined parameters [45,46]. The survey comprised 11 questions overall, and the questionnaire was pilot-tested in a small sample (n=15) of cardiac patients similar to the sample. Moreover, the content of the questionnaire was reviewed and amended to improve the ease of use, accuracy, and specificity. Furthermore, the questionnaire was used in a larger study including 282 English-speaking patients [39].

Questions regarding each device were clarified using an illustration (Textbox 1). Most questions (questions 1, 3-9) were in the checklist format, where respondents ticked the technological devices or features that they (1) used, (2) used confidently, (3) would like to learn, and (4) used for health purposes. In addition, we developed a question (question 2) on self-efficacy in using a new computer program based on the speed with which participants could learn a new computer program and used a 4-point scale anchored with responses “very slowly” (1) and “very quickly” (4). Furthermore, we included two open-ended questions (questions 10 and 11) where respondents could provide additional information on the mobile app they used along with details of the health-related app. Notably, respondents who answered the first question with “not using any technology” were not required to complete the remainder of the questionnaire.

Finally, the questionnaire was translated into Mandarin by a certified translator and back-translated for verification. A minor amendment was made to item seven that inquired about the videoconferencing use—WeChat or QQ was surveyed instead of Skype or FaceTime because it was more popular in Mandarin-speaking communities.

### Procedure

The study protocol was approved by Northern Sydney Local Health District Human Research Ethics Committee (LNR/15/HAWKE/450). All patients were screened for the eligibility by a CR staff (a clinical nurse specialist) or the bilingual researcher (LZ) upon their admission to a cardiac ward of the hospital or upon referral to the outpatient CR programs at the study site between April and September 2016. Those who fulfilled the inclusion criteria were approached by the CR staff or LZ to participate in the study and provided them with information and time to consider participation. Mandarin-speaking patients were approached by a bilingual Mandarin-speaking CR staff member or LZ. All staff members were trained in using the questionnaire to ensure a standardized approach. Finally, patients who provided written consent were surveyed in this study. The CR staff or LZ collected demographic and clinical data of enrolled patients, and any uncertainty regarding diagnoses was clarified using the medical records. Notably, the questionnaire was self-administered. Of 134 CR patients approached, 10 declined because of the lack of interest, with the final response rate of 92.5%. We surveyed 94 patients from English and “other” language-speaking background for the ongoing matching purpose; of these, 60 participants were matched and enrolled in the final data analysis.

### Statistical Analyses

The responses in Mandarin were translated into English by LZ for data entry. Data analyses were performed using IBM SPSS, version 24. In addition, means, SDs, frequencies, and percentage were used to present the demographic and clinical characteristics of the study cohort. Frequencies and percentages were used to describe technology device and feature use, confidence in use, and use for health. Furthermore, categorical variables were reported as a percentage within a language group and tested for differences across language groups using chi-square tests.

We used generalized linear mixed model analysis (GLMM) to ascertain whether the language spoken at home correlated with the mobile device and feature use. In addition, GLMM was used as patients in each language group were selected to be matched for age and gender. Each group of three (one from each language group) was assigned the same group identification (ID; 30 in all) besides a unique individual ID. As outcomes (ie, whether specific types of technology or features were used) were dichotomous, we selected the binary logistic regression function.

**Technology questionnaire.**

1. Which of the following do you currently use?

Computers, Tablets, Mobile phones, Smartphones, Activity trackers, None

2. How quickly can you work out how to use new computer programs? Select one answer.

Very slowly, Fairly slowly, Fairly quickly, Very quickly

For the following questions apply to:

Computers, Tablets, Mobile phones, Smartphones, Activity trackers, None

3. I feel confident using these devices:

4. I share health information through these devices:

5. I do not use these devices but would like to learn:

6. I think I could easily learn how to use these devices:

7. What do you regularly use your mobile/smartphone or tablet for?

Voice calls, Text messages, Skype or FaceTime (WeChat or QQ), Browsing the internet, Checking emails, Social media, Schedule/calendar, Using mobile apps

8. Do you use the internet for accessing information on any of the following?

Health conditions, Medication, Heart conditions, Heart treatments, Lifestyle changes, Health resources

9. Do you use the internet for communicating with?

Health professionals, other heart patients

10. How many apps are currently on your phone?

11. Please list any health-related apps you use:

Adjusted models comprised age, gender, years of education, marital status, and employment status in the model along with language spoken at home. Furthermore, we explored devices and mobile features that were reported the most prevalent in participants’ report or those that had the highest potential for CR interventions. Then, we assessed correlates of using devices (ie, smartphone, computer, and tablet) and mobile features (ie, the internet, emails, apps, and social media). Finally, the internet use for health was categorized into individual items, including sharing health information, accessing information about general health, medication, and lifestyle. In this study, odds ratios (OR), 95% CI, and *P* values are reported, and alpha=0.05. *P* ≤.05 was considered statistically significant (two-tailed).

## Results

### Descriptive Statistics

In this study, the final sample comprised 90 patients (mean age 66.7, SD 13 years; range 46-95 years); of these, 53.3% (48/90) were males, 55.6% (50/90) completed high school, and 63.3% (57/90) were not in the workforce. More than half of the participants were admitted with CHD (52/90, 57.7%), with the leading procedures or diagnoses being PCI, angina, and MI (Table 1). In the “other” language group, the most common languages spoken at home were Greek (7/30, 23.3%), Arabic (6/30, 20%), and the remainder comprised Macedonian, Vietnamese, Hungarian, Italian, Russian, Indonesian, Portuguese, Philippine, Japanese, Samoa, French, Bulgarian, and Czech language. We observed no significant difference in education, marital status, living arrangement, and employment status across the three home language groups.

### Use of Mobile Technology by the Home Language Group

**Figure 1:**
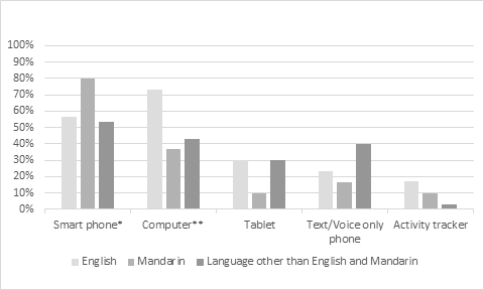
Technology device use by home language group.*Mandarin-speaking group vs Language other than English and Mandarin speaking group (*P*=.03); **English-speaking group vs Mandarin-speaking group (*P*=.003); English-speaking group vs Language other than English and Mandarin speaking group (*P*=.02).

Most participants (82/90, 91.1%) reported using, at least, one of the following devices: computers (desktops or laptops), tablets, smartphones, text/voice-only phones, and activity trackers (Figure 1). Mobile devices, such as tablets, smartphones, text/voice-only phones, and activity trackers, were used by most participants(n=79, 87.8%), the most common of which were smartphones (57/90, 63.3%), followed by tablets (28/90, 31.1%), and text/voice-only phones (24/90, 26.7%). In addition, 33.3% (8/24) of text/voice-only phone users displayed their interest in learning to use a smartphone in the future. The mean score on how quickly one could learn a new computer program was 2.16 (SD 1.0), with 1 representing “very slowly,” 2 representing “fairly slowly,” 3 representing “fairly quickly,” and 4 representing “very quickly,” suggesting that the participants on average could learn a new computer program but might require time.

In this study, the three language groups were similar in the mobile technology use, except for the smartphone use. The proportion of smartphone users in the Mandarin-speaking group was significantly higher compared with “other” language-speaking group (80.0% vs 53.3%; *P*=.03). The confidence in the current mobile technology use was similar across groups, except for the confidence in using text/voice-only phones. A larger proportion of participants in the “other” language-speaking group were only confident in using text/voice-only phones compared with the Mandarin-speaking group (51.9% vs 17.2%; *P*=.006).

**Table 1:** Sample characteristics and technology use compared by the home language group.

Characteristics		Overall	English^a^	Mandarin^b^	Other^c^	*P*
Age (mean, SD)		66.7 (13.1)	66.6 (13.7)	66.9 (13.9)	66.4 (12.0)	.99
Gender (male), n (%)		48 (53.3)	16 (53.3)	16 (53.3)	16 (53.3)	>.99
Completed high school, n (%)		50 (55.6)	15 (50.0)	19 (63.3)	16 (53.3)	.56
Employed, n (%)		33 (36.7)	13 (43.3)	9 (30.0)	11 (36.7)	.56
Married or partner, n (%)		62 (68.9)	20 (66.7)	24 (80.0)	18 (60.0)	.39
Living with family, n (%)		75 (83.3)	23 (76.7)	29 (96.7)	23 (76.7)	.06
Admitted with CHD^d^, n (%)		52 (57.7)	16 (53.3)	17 (56.7)	19 (63.3)	.65
**Technology use**						
	Mobile technology^e^, n (%)	79 (87.8)	23 (76.7)	29 (96.7)	27 (90.0)	.06
	Mobile apps, n (%)	42 (46.7)	18 (69.2)	14 (48.3)	10 (37.0)	.06
Learn a new computer program (1—lowest, 4—highest), mean (SD)		2.16 (1.00)	2.54 (0.99)	1.97 (0.91)	2.00 (1.04)	.06

^a^English-speaking group.

^b^Mandarin-speaking group.

^c^Language other than English and Mandarin.

^d^CHD: coronary heart disease.

^e^The mobile technology includes tablets, smartphones, text- or voice-only phones, and activity trackers.

**Figure 2:**
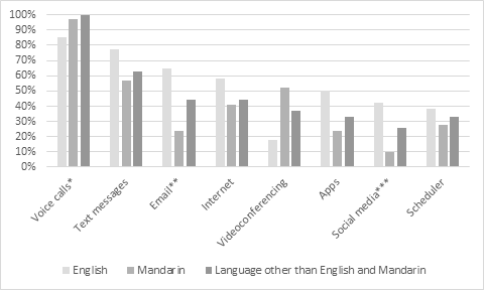
Technology feature use by home language group. *English-speaking group vs Language other than English and Mandarin speaking group (*P*=.05); **English-speaking group vs Mandarin-speaking group (*P*=.001); *** English-speaking group vs Mandarin-speaking group (*P*=.007).

However, computer use significantly differed across home language groups, with more English-speaking participants using computer compared with Mandarin or “other” language-speaking participants (English: 73.3% vs Mandarin: 36.7%, *P*=.003; English: 73.3% vs “other” language: 43.3%; *P*=.02). Furthermore, the proportion of participants confident in using a computer was significantly higher in the English-speaking group compared with the Mandarin-speaking groups (73.1% vs 35%; *P*=.003).

### Use of Mobile Features by Language Group

Mobile features most commonly used among participants using mobile device were voice calls (77/82, 93.9%), text messages (54/82, 65.9%), the internet (39/82, 47.6%), emails (36/82, 43.9%), videoconferencing (Skype or FaceTime [WeChat or QQ]; 35/82, 42.7%; Figure 2). In addition, fewer Mandarin-speaking participants used emails (24.1% vs 65.4%; *P*=.001) and social media (10.3% vs 42.3%; *P*=.007) compared with English-speaking participants.

**Figure 3:**
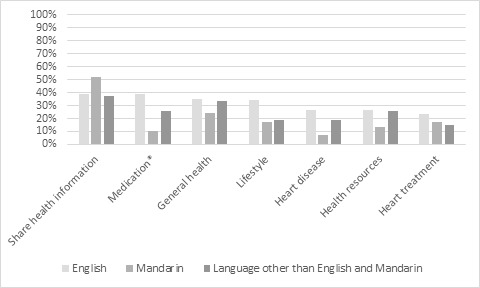
Internet use for health purposes by home language group.*English-speaking group vs Mandarin-speaking group (*P*=.02).

Overall, 44.4% (36/81) of the participants who engaged with technology used the internet for health (Figure 3), used most often for sharing health information (35/81, 42.7%) and accessing information about general health (25/81, 30.5%), medication (20/81, 24.4%), and lifestyle (19/81, 23.2%). We observed no significant difference across groups in using the internet for health, except that a higher percentage of English-speaking participants accessed the web-based medication information than Mandarin-speaking participants (38.4% vs 10.4%; *P*=.02).

### Correlates of Using Mobile Devices and Features

After adjusting for age, gender, years of education, marital status, and employment status, Mandarin-speaking participants exhibited increased odds of using smartphones (OR 7.238, 95% CI 1.262-41.522; *P*=.03) but decreased odds of using emails (OR 0.089, 95% CI 0.016-0.490; *P*=.006), and accessing the web-based medication information (OR 0.191, 95% CI 0.037-0.984; *P*=.05) compared with English-speaking participants (Tables 2 and 3). In addition, other factors associated with mobile devices and features use; for an additional year in age, the odds of using smartphones and emails decreased (OR 0.118, 95% CI 0.809-0.961; *P*=.005; OR 0.104, 95% CI 0.820-0,978; *P*=.02). Furthermore, participants who were employed exhibited increased odds of using Apps and social media compared with their nonworking counterparts (OR 6.052, 95% CI 1.256-29.175; *P*=.03; OR 16.455; *P*=.01). Male participants exhibited decreased odds of using a tablet compared with females (OR 0.163, 95% CI 0.044-0.600; *P*=.007).

**Table 2:** Correlates of using technological devices, based on logistic regression models mutually adjusted for all variables listed in the table.

Characteristic		Smartphones			Computers			Tablets		
		OR^a^	95% CI	*P*	OR	95% CI	*P*	OR	95% CI	*P*
Age		0.882	0.809-0.961	.005	0.979	0.919-1.043	.51	0.974	0.913-1.039	.42
Gender (male)		0.618	0.141-2.703	.52	0.949	0.271-3.319	.93	0.163	0.044-0.600	.007
Years of education		1.093	0.935-1.278	.26	1.163	1.001-1.350	.05	1.122	0.973-1.295	.11
Married		3.258	0.757-14.018	.11	3.583	0.863-14.874	.08	2.64	0.685-10.175	.16
Employed		2.114	0.247-18.106	.49	7.537	1.366-41.602	.02	2.718	0.542-13.629	.22
**Language**										
	Mandarin vs English^b^	7.238	1.262-41.522	.03	0.120	0.027-0.546	.007	1.361	0.371-4.991	.64
	Other vs English^c^	0.948	0.200-4.490	.95	0.223	0.051-0.974	.05	1.282	0.344-4.772	.71

^a^OR: odds ratio.

^b^Mandarin-speaking group vs English-speaking group.

^c^Language other than English vs Mandarin-speaking group.

**Table 3:** Correlates of using mobile features, based on logistic regression models mutually adjusted for all variables listed in the table.

Characteristic		Internet			Emails			Apps			Social media		
		OR^a^	95% CI	*P*	OR	95% CI	*P*	OR	95% CI	*P*	OR	95% CI	*P*
Age		0.968	0.904-1.035	.33	0.896	0.820-0.978	.02	0.960	0.888-1.037	.30	0.947	0.861-1.042	.26
Gender (male)		1.228	0.357-4.230	.74	0.504	0.120-2.114	.34	1.239	0.340-4.521	.74	0.368	0.071-1.911	.23
Years of education		1.034	0.897-1.193	.64	1.106	0.954-1.283	.18	1.059	0.916-1.226	.43	0.956	0.808-1.131	.59
Married		1.915	0.477-7.696	.36	2.146	0.470-9.804	.32	2.634	0.595-11.662	.20	0.862	0.174-4.276	.85
Employed		4.332	0.897-20.924	.07	2.519	0.487-13.019	.27	6.052	1.256-29.175	.03	16.455	1.937-139.767	.01
**Language**													
	Mandarin vs English^b^	0.579	0.140-2.397	.45	0.089	0.016-0.490	.006	0.297	0.065-1.354	.12	0.199	0.035-1.121	.07
	Other vs English^c^	0.653	0.160-2.658	.55	0.345	0.075-1.588	.17	0.557	0.137-2.274	.41	0.529	0.123-2.285	.39

^a^OR: odds ratio.

^b^Mandarin-speaking group vs English-speaking group.

^c^Language other than English vs Mandarin-speaking group.

## Discussion

### Principal Findings

To the best of our knowledge, this is the first exploratory study on the mobile technology use among CALD patients in the CR setting. Overall, the study suggests that technology might provide an alternative secondary prevention delivery strategy in the future to bridge the gap between growing demands and limited resources, as population aging and CVD prevalence continues to rise. Given the increasing use of technology-based interventions in CVD secondary prevention, this study reveals the unique patterns of use among CALD patients. In addition, the findings indicate that a variation in technology use warrants consideration while developing or delivering technology-based CR to these groups. The study determined that CALD patients are not disadvantaged in using certain types of mobile technology; thus, technology-based interventions could offer a potential solution to overcome their barriers to attending CR, such as communication and transportation difficulties. Meanwhile, the technology use patterns among the study groups revealed that selecting appropriate delivery media is essential for reaching different patients groups to improve the CR uptake.

Although several CALD patients are non-computer users, possibly because they had few opportunities to acquire computer skills during their education and work [47,48], they are not disadvantaged in some mobile technology use, especially not in the smartphone use. Consistently, the smartphone ownership is the highest among CALD groups [49], offering a great promise for implementing smartphone-based interventions in these populations. An important principle for adapting health promotion interventions in CALD populations is to determine and address the barriers to access and participation to decrease disparities [50]. Traditionally, patients from CALD backgrounds have been identified among those who are least likely to attend CR programs [51], especially if they do not speak English, do not drive a car, have lower education or income, or have cultural barriers such as embarrassment of participation [14,21,23,51]. Technology-based CR could potentially address these barriers, as the program can be adapted to different languages and is not constrained by facilities, transportation, and time. Furthermore, it can be used in a patient-preferred environment [51] to improve the patient’s participation, engagement, and overall experience [38].

The variation in the mobile technology use among CALD groups warrants elucidation and accommodation when developing or delivering these interventions. In addition, evaluating the usage of mobile technology before developing or delivering to the targeted population is imperative. For instance, no overall significant difference has been reported in the internet use for health between CALD patients and others in this study, implying that internet-based interventions could potentially reach CALD as well as English-speaking patients. Evidence from this and other studies suggests that people from CALD backgrounds tend to access the internet more by smartphones rather than computers or laptops [49]. Thus, internet-based programs should be user-friendly for both computer and smartphone users to encourage participation. Furthermore, smartphone users use mobile features differently. For example, Mandarin-speaking patients tend to use emails less compared with English-speaking patients. Thus, email-based communication in CVD secondary prevention might not be feasible among certain CALD groups [47]. Reportedly, selecting an appropriate delivery method for CR interventions is the key to improving participation among CALD patients [52].

Overall, the ubiquity of mobile technology could potentially enable technology-based interventions in the future to fulfill the increasing demand for CR in CALD patients. Presumably, the population aged >60 years will increase from the current 800 million (representing 11% of the world population) to >2 billion in 2050 (representing 22% of the world population) [53], which would result in a tremendous challenge for health care in dealing with the increasingly prevalent noncommunicable diseases such as CHD. Meanwhile, mobile technology ownership has also witnessed an exponential upsurge [54,55]. Unlike the initial digital divide that placed the computer use and internet access beyond the reach of many older and lower-income individuals, the mobile technology has been extensively adopted across populations [54,56]. A well-established interest in technology enabled CR [57,58], implies that this new form of intervention and delivery might provide an alternative to meet the increasing demands [59]. In addition, some preliminary evidence suggest that technology-based CR has the potential for cost-saving compared with center-based CR [37,38]. However, age-related differences in the mobile feature use suggest that voice call- and text message-based interventions could be superior in reaching current older CR patients [57,58]. Furthermore, apps- and social media-based interventions could have great potential for future CR delivery when young users of today become tomorrow’s CR patient population [58].

### Limitations

This study has several limitations. First, the predetermined sample size is relatively small, and the sample was enrolled from a single hospital in Australia, which might limit the generalizability of the findings to the larger CR population. However, this study does provide crucial insights into the future research. Second, this study primarily aimed to assess the role of Mandarin as a home language in the technology use compared with other language groups. Given that English language users experience different challenges to non-English language users, we recruited two samples to compare with Mandarin-speaking patients. The “other” language-speaking group provides scope for comparison of the effects of a home language other than English that contrast Mandarin speakers. In addition, language spoken at home might be a marker for people’s acculturation level and English language proficiency, which were not assessed. Further studies are required to investigate the subgroups of language and cultures within this diverse group. Furthermore, any differences identified might represent the CALD experience in Australia and, thus, might not be able to be extrapolated to CALD populations in other countries. Third, we did not correct alpha for multiple pairwise comparisons and acknowledge the risk of type I errors because of the exploratory nature of the study and the small sample size. Finally, data collection using self-administered questionnaires is subject to recall and social desirability biases. Hence, further studies should complement self-administered questionnaire with objective measures and in-depth investigation of the role of home language and other correlates of technology use.

### Conclusions

This study reveals a high usage of mobile technology among CR patients and provides further insights into differences in technology use across CALD patients in Australia. The findings of this study could be used to guide the design and implementation of the technology-based CR. Furthermore, mobile technology-based CR interventions seem promising to patients from CALD backgrounds, and the identification of the relevant technology use is the key to a successful implementation.

## Abbreviations

CABG: coronary artery bypass graft
CALD: culturally and linguistically diverse
CHD: coronary heart disease
CR: cardiac rehabilitation
GLMM: generalized linear mixed model
IHD: ischemic heart disease
MI: myocardial infarction
PCI: percutaneous coronary intervention

## References

[1] Bansilal S, Castellano JM, Fuster V (2015). Global burden of CVD: focus on secondary prevention of cardiovascular disease. Int J Cardiol.

[2] Jernberg T, Hasvold P, Henriksson M, Hjelm H, Thuresson M, Janzon M (2015). Cardiovascular risk in post-myocardial infarction patients: nationwide real world data demonstrate the importance of a long-term perspective. Eur Heart J.

[3] Brown TM, Deng L, Becker DJ, Bittner V, Levitan EB, Rosenson RS, Safford MM, Muntner P (2015). Trends in mortality and recurrent coronary heart disease events after an acute myocardial infarction among Medicare beneficiaries, 2001-2009. Am Heart J.

[4] Anderson L, Taylor RS (2014). Cardiac rehabilitation for people with heart disease: an overview of Cochrane systematic reviews. Cochrane Database Syst Rev.

[5] Lawler PR, Filion KB, Eisenberg MJ (2011). Efficacy of exercise-based cardiac rehabilitation post-myocardial infarction: a systematic review and meta-analysis of randomized controlled trials. Am Heart J.

[6] Pasquali SK, Alexander KP, Peterson ED (2001). Cardiac rehabilitation in the elderly. Am Heart J.

[7] Taylor RS, Brown A, Ebrahim S, Jolliffe J, Noorani H, Rees K, Skidmore B, Stone JA, Thompson DR, Oldridge N (2004). Exercise-based rehabilitation for patients with coronary heart disease: systematic review and meta-analysis of randomized controlled trials. Am J Med.

[8] Anderson L, Oldridge N, Thompson DR, Zwisler A, Rees K, Martin N, Taylor RS (2016). Exercise-Based Cardiac Rehabilitation for Coronary Heart Disease: Cochrane Systematic Review and Meta-Analysis. J Am Coll Cardiol.

[9] Canyon S, Meshgin N (2008). Cardiac rehabilitation - reducing hospital readmissions through community based programs. Aust Fam Physician.

[10] Heran BS, Chen JM, Ebrahim S, Moxham T, Oldridge N, Rees K, Thompson DR, Taylor RS (2011). Exercise-based cardiac rehabilitation for coronary heart disease. Cochrane Database Syst Rev.

[11] Sagar VA, Davies EJ, Briscoe S, Coats AJS, Dalal HM, Lough F, Rees K, Singh S, Taylor RS (2015). Exercise-based rehabilitation for heart failure: systematic review and meta-analysis. Open Heart.

[12] Pack QR, Squires RW, Lopez-Jimenez F, Lichtman SW, Rodriguez-Escudero JP, Zysek VN, Thomas RJ (2014). The current and potential capacity for cardiac rehabilitation utilization in the United States. J Cardiopulm Rehabil Prev.

[13] Pavy B, Darchis J, Merle E, Caillon M (2014). [Cardiac rehabilitation after myocardial infarction in France: still not prescribed enough]. Ann Cardiol Angeiol (Paris).

[14] Neubeck L, Freedman SB, Clark AM, Briffa T, Bauman A, Redfern J (2012). Participating in cardiac rehabilitation: a systematic review and meta-synthesis of qualitative data. Eur J Prev Cardiol.

[15] De VC, Li X, Van VI, Saka O, Dendale P, Eyssen M, Paulus D (2013). Participating or not in a cardiac rehabilitation programme: factors influencing a patient's decision. Eur J Prev Cardiol.

[16] Clark AM, King-Shier KM, Duncan A, Spaling M, Stone JA, Jaglal S, Angus J (2013). Factors influencing referral to cardiac rehabilitation and secondary prevention programs: a systematic review. Eur J Prev Cardiol.

[17] Clark AM, King-Shier KM, Spaling MA, Duncan AS, Stone JA, Jaglal SB, Thompson DR, Angus JE (2013). Factors influencing participation in cardiac rehabilitation programmes after referral and initial attendance: qualitative systematic review and meta-synthesis. Clin Rehabil.

[18] Cooper AF, Jackson G, Weinman J, Horne R (2002). Factors associated with cardiac rehabilitation attendance: a systematic review of the literature. Clin Rehabil.

[19] Scott LAB, Ben-Or K, Allen JK (2002). Why are women missing from outpatient cardiac rehabilitation programs? A review of multilevel factors affecting referral, enrollment, and completion. J Womens Health (Larchmt).

[20] Soo HSY, Gallagher R, Elliott D (2016). Predictors of cardiac rehabilitation attendance following primary percutaneous coronary intervention for ST-elevation myocardial infarction in Australia. Nurs Health Sci.

[21] Jolly K, Lip GY, Taylor RS, Mant JW, Lane DA, Lee KW, Stevens AJ, BRUM Steering Committee (2005). Recruitment of ethnic minority patients to a cardiac rehabilitation trial: the Birmingham Rehabilitation Uptake Maximisation (BRUM) study [ISRCTN72884263]. BMC Med Res Methodol.

[22] Valencia HE, Savage PD, Ades PA (2011). Cardiac rehabilitation participation in underserved populations. Minorities, low socioeconomic, and rural residents. J Cardiopulm Rehabil Prev.

[23] Chauhan U, Baker D, Lester H, Edwards R (2010). Exploring uptake of cardiac rehabilitation in a minority ethnic population in England: a qualitative study. Eur J Cardiovasc Nurs.

[24] Mead H, Ramos C, Grantham SC (2016). Drivers of Racial and Ethnic Disparities in Cardiac Rehabilitation Use: Patient and Provider Perspectives. Med Care Res Rev.

[25] (2017). Australian Bureau of Statistics.

[26] (2017). Australian Bureau of Statistics.

[27] Jin K, Ding D, Gullick J, Koo F, Neubeck L (2015). A Chinese Immigrant Paradox? Low Coronary Heart Disease Incidence but Higher Short-Term Mortality in Western-Dwelling Chinese Immigrants: A Systematic Review and Meta-Analysis. J Am Heart Assoc.

[28] Zhang L, Gallagher R, Ding D, Neubeck L (2018). Self-management Following a Cardiac Event in People of Chinese Ethnicity Living in Western Countries: A Scoping Review. J Immigr Minor Health.

[29] Pottie K, Batista R, Mayhew M, Mota L, Grant K (2014). Improving delivery of primary care for vulnerable migrants: Delphi consensus to prioritize innovative practice strategies. Can Fam Physician.

[30] Abbott MW, Wong S, Williams M, Au MK, Young W (2000). Recent Chinese migrants' health, adjustment to life in New Zealand and primary health care utilization. Disabil Rehabil.

[31] Liu C, Ingleby D, Meeuwesen L (2011). Barriers to health care for chinese in the Netherlands. Int J Family Med.

[32] Martínez-Pérez B, de LTI, López-Coronado M, Herreros-González J (2013). Mobile apps in cardiology: review. JMIR Mhealth Uhealth.

[33] Cajita MI, Gleason KT, Han H (2016). A Systematic Review of mHealth-Based Heart Failure Interventions. J Cardiovasc Nurs.

[34] Neubeck L, Redfern J, Fernandez R, Briffa T, Bauman A, Freedman SB (2009). Telehealth interventions for the secondary prevention of coronary heart disease: a systematic review. Eur J Cardiovasc Prev Rehabil.

[35] Dithmer M, Rasmussen JO, Grönvall E, Spindler H, Hansen J, Nielsen G, Sørensen SB, Dinesen B (2016). “The Heart Game”: Using Gamification as Part of a Telerehabilitation Program for Heart Patients. Games Health J.

[36] Neubeck L (2015). Telehealth-based cardiac rehabilitation: A solution to the problem of access?. Eur J Prev Cardiol.

[37] Whittaker F, Wade V (2014). The costs and benefits of technology-enabled, home-based cardiac rehabilitation measured in a randomised controlled trial. J Telemed Telecare.

[38] Kairy D, Lehoux P, Vincent C, Visintin M (2009). A systematic review of clinical outcomes, clinical process, healthcare utilization and costs associated with telerehabilitation. Disabil Rehabil.

[39] Gallagher R, Roach K, Sadler L, Glinatsis H, Belshaw J, Kirkness A, Zhang L, Gallagher P, Paull G, Gao Y, Partridge SR, Parker H, Neubeck L (2017). Mobile Technology Use Across Age Groups in Patients Eligible for Cardiac Rehabilitation: Survey Study. JMIR Mhealth Uhealth.

[40] (2014). NSW Health: South Eastern Local Health District.

[41] (2014). NSW Health: South Eastern Local Health District.

[42] Feltbower RG, Fleming SJ, Picton SV, Alston RD, Morgan D, Achilles J, McKinney PA, Birch JM (2014). UK case control study of brain tumours in children, teenagers and young adults: a pilot study. BMC Res Notes.

[43] Wilson Van Voorhis CR, Morgan BL (2007). Understanding Power and Rules of Thumb for Determining Sample Sizes. TQMP.

[44] Gallagher R, Roach K, Sadler L, Belshaw J, Kirkness A, Zhang L, Proctor R, Neubeck L (2015). Who gets stroke prevention? Stroke prevention in atrial fibrillation patients in the inpatient setting. Heart Lung Circ.

[45] Edwards L, Thomas C, Gregory A, Yardley L, O'Cathain A, Montgomery AA, Salisbury C (2014). Are people with chronic diseases interested in using telehealth? A cross-sectional postal survey. J Med Internet Res.

[46] Illiger K, Hupka M, von JU, Wichelhaus D, Albrecht U (2014). Mobile technologies: expectancy, usage, and acceptance of clinical staff and patients at a university medical center. JMIR Mhealth Uhealth.

[47] González A, Ramírez MP, Viadel V (2015). ICT Learning by Older Adults and Their Attitudes toward Computer Use. Curr Gerontol Geriatr Res.

[48] Newman L, Biedrzycki K, Baum F (2012). Digital technology use among disadvantaged Australians: implications for equitable consumer participation in digitally-mediated communication and information exchange with health services. Aust Health Rev.

[49] Burke LE, Ma J, Azar KMJ, Bennett GG, Peterson ED, Zheng Y, Riley W, Stephens J, Shah SH, Suffoletto B, Turan TN, Spring B, Steinberger J, Quinn CC, American HAPCOTCOEBCCOTCOCHCOCNCOFGBCOQOCRC (2015). Current Science on Consumer Use of Mobile Health for Cardiovascular Disease Prevention: A Scientific Statement From the American Heart Association. Circulation.

[50] Netto G, Bhopal R, Lederle N, Khatoon J, Jackson A (2010). How can health promotion interventions be adapted for minority ethnic communities? Five principles for guiding the development of behavioural interventions. Health Promot Int.

[51] Jolly K, Greenfield SM, Hare R (2004). Attendance of ethnic minority patients in cardiac rehabilitation. J Cardiopulm Rehabil.

[52] Clark RA, Conway A, Poulsen V, Keech W, Tirimacco R, Tideman P (2015). Alternative models of cardiac rehabilitation: a systematic review. Eur J Prev Cardiol.

[53] Bloom D, Boersch-Supan A, McGee (2011). AARP.

[54] (2018). Australian Bureau of Statistics.

[55] Australian Bureau of Statistics.

[56] Smith A (2015). Pew Research Center: Internet and Technology.

[57] Dale LP, Whittaker R, Eyles H, Mhurchu CN, Ball K, Smith N, Maddison R (2014). Cardiovascular Disease Self-Management: Pilot Testing of an mHealth Healthy Eating Program. J Pers Med.

[58] Buys R, Claes J, Walsh D, Cornelis N, Moran K, Budts W, Woods C, Cornelissen VA (2016). Cardiac patients show high interest in technology enabled cardiovascular rehabilitation. BMC Med Inform Decis Mak.

[59] Pietrzak E, Cotea C, Pullman S (2014). Primary and secondary prevention of cardiovascular disease: is there a place for Internet-based interventions?. J Cardiopulm Rehabil Prev.

